# The complete mitochondrial genome and phylogenetic analysis of the Arabian Honeybee *Apis mellifera jemenitica* (Insecta: Hymenoptera: Apidae) from Saudi Arabia

**DOI:** 10.1080/23802359.2020.1832598

**Published:** 2020-11-03

**Authors:** Ahmad Alghamdi, Yehya Alattal

**Affiliations:** Eng. Abdullah Bagshan for Bee Research, College of Food and Agriculture Sciences, King Saud University, Riyadh, Saudi Arabia

**Keywords:** Mitogenomes, NGS, *Apis mellifera jemenitica*, phylogeny

## Abstract

Fourteen mitochondrial genomes from workers of the Arabian Honeybee *Apis mellifera jemenitica* were determined. Genomes range from 16,352 to 16,445 bp. Each consists of 13 protein-coding genes, 22 transfer RNAs, two ribosomal RNAs, and one control region. The mitogenome sequences revealed 753 Variable sites in total, distributed within protein-coding loci (199), ribosomal RNAs genes (117), transfere RNAs genes (48), and non-coding AT-rich region's (389). Phylogenetic analysis with Neighbor-Joining method suggested three evolutionary groups for these mitogenomes, closely related to *A. m. jemenitica*, *A. m. lamarckii*, *A. m. syriaca,* and *A. m. scutellata*.

## Introduction

Throughout the Arabian Peninsula, it is estimated that the Arabian honeybee, *A. m. jemenitica*, has been used in apiculture for more than 4000 years (Alqarni et al. 2011). In western and central Saudi Arabia where temperature extremes for the Arabian Peninsula pertains, only native *A. m. jemenitica* can survive, while other standard subspecies fail to persist (Alghamdi et al. 2013; Alattal and Alghamdi 2015). A previous study based on morphological traits separated honeybee population within Saudi Arabia into three distinctive subpopulations, with a cline in body size from far north to far south of Saudi Arabia (Alghamdi et al. 2012), which may reflect specific evolutionary relationships among proposed subpopulations. Here we present the complete mitogenomes of 14 *A. m. jemenitica* workers collected in 2020 from native, non-migratory and feral colonies from Saudi Arabia and document the evolutionary relationship among them.

## Methods

Genomic DNA was extracted using Qiagen DNeasy Blood and Tissue Kit (Cat No./ID: 69506) and then sequenced using Illumina Hi-Seq system. Pair-end short reads (150 bp) DNA libraries were sequenced by BGI (Hong Kong, China) and filtered using Flexbar v3.4.0 (Roehr et al. 2017). Trimmed reads were mapped and annotated in Geneious Prime 2020.1.2 (Biomatters Ltd., Auckland, New Zealand) using the mitogenome of *A. m. jemenitica* (GeneBank: MN714161), for a sample collected from Yemen in 1988 (Boardman et al. 2020) as a reference. Sequences were aligned to other *Apis* mitogenomes using MEGA7 (Kumar et al. 2016). Phylogenetic tree reconstruction was performed on PCGs of the complete genome sequences in Mega7 using Neighbor-Joining-Maximum Composite Likelihood method and tested over 1000 bootstrap replicates (Felsenstein 1985), evolutionary distances as the number of base substitutions per site were calculated in MEGA7 (Kumar et al. 2016).

## Results and discussion

**Figure 1.**
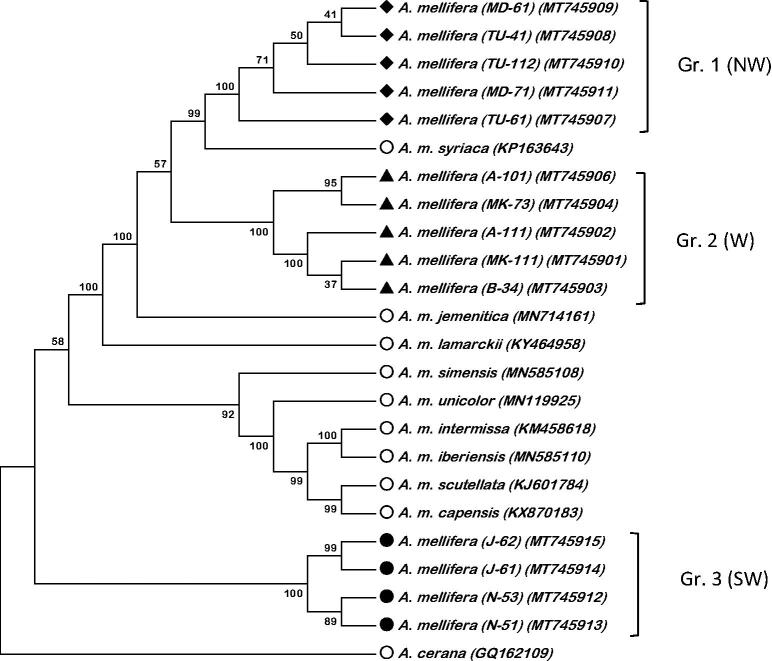
Phylogenetic tree using Neighbor-Joining method showing the relationship between Arabian Honeybee, *Apis mellifera*, mitogenomes and nine other *Apis* honeybee mitogenomes. The tree is midpoint rooted, node labels indicate bootstrap values. N = North, S = South and W = West parts of Saudi Arabia. The symbols in the first bracts after the species name resemble the sampling location and sample code; A: Asir; B; Baha; J: Jazan; MD: Madinah; MK; Makkah; TU: Tabuk.

The complete mitogenomes reported here (MT745901; MT745902; MT745903; MT745904; MT745906; MT745907; MT745908; MT745909; MT745910; MT745911; MT745912; MT745913; MT745914; MT745915) range from 16,352-16,445 bp. The average proportions of the four nucleotides in the mitogenomes were 43.26 ± 0.03 for adenine (A), 9.62 ± 0.01 for cytocine (C), 5.57 ± 0.02 for guinine (G) and 41.53 ± 0.03 for thymine (T). Each mitogenome has 13 protein-coding genes (PCGs), two ribosomal (rRNA) genes, and 22 transfer RNA (tRNA) genes. Nine of the 13 PCGs were located on the light strand and four on the heavy strand. Six PCGs started with ATT, four started with ATG, two with ATA, and one with ATC. All PCGs ended with a TAA stop codon. Of the 22 tRNAs identified the shortes was 63 bp (tRNA-Gln) and had the same length in all mitogenomes, while the longest ranged between 78 and 80 bp (tRNA-Thr). The two rRNA strand ranged from 788 to 791 bp (12S) and 1338 to 1367 bp (S16). In total, variable sites were 753; 48 for tRNAs, 117 for rRNAs, 199 for protein-coding regions (PCGs) and 389 for non-coding AT-rich regions. Our mitogenomes show high similarity with earlier reported mitogenome (MN714161) of *A. m. jemenitica*, yet revealed three related groups (Figure 1), group one and two are very closely related (Evolutionary distance (*p*) = 0.003), group three show a distance (*p*) of 0.017 and 0.018 with group one and two respectively. Group one is closest to *A. m. syriaca* (*p* = 0.002), though group two is very close to *A. m. jemenetica* and *A. m. lamarckii* (*p* = 0.003–0.004), respectively. Group three is different and is closer to African honeybee subspecies (nearest is *A. m. scutellata* (*p* = 0.016)). Among reference mitogenomes, evolutionary distance between *A. m. syriaca* and *A. m. scutellata* is as low as 0.012. Apparently, these mitogenome sequences would enrich our understanding of evolutionary relationships among the Middle East and African honeybee subspecies.

## Geolocation information

Samples geospatial coordinates ((MT745901): 26°.16′S:37°.45′E; (MT745902):22°.17′S:41°.40′E; (MT745903):24°.93′S:37°.73′E; (MT745904):24°.34′S:38°.95′E; (MT745906):26°.16′S:37°.45′E; (MT745907):21°.19′S:39°.59′E; (MT745908):18°.25′S:42°.23′E; (MT745909):18°.25′S:42°.23′E; (MT745910):19°.85′S:41°.58′E; (MT745911):21°.91′S:39°.75′E; (MT745912):17°.49′S:44°14′.E; (MT745913):17°.49′S:44°.14′E; (MT745914):17°.51′S:43°.07′E; (MT745915): 17°.33′S: 43°.03′E). Specimens are disposed at King Saud University Insect Museum.

## Data Availability

The data that support the findings of this study are openly available in the Gene Bank [NCBI] at [https://www.ncbi.nlm.nih.gov], reference number [MT745901-MT745904 and MT745906-MT745915].
